# Comparison of cubesat and microsat catastrophic failures in function of radiation and debris impact risk

**DOI:** 10.1038/s41598-022-27327-z

**Published:** 2023-01-07

**Authors:** Isabel Lopez-Calle, Alexander I. Franco

**Affiliations:** Department of Electronics and System Engineering, University of Cadiz, 11519 Puerto Real, Spain

**Keywords:** Environmental impact, Aerospace engineering, Electrical and electronic engineering

## Abstract

Occurrence level comparison of catastrophic failures due to radiation effects on electronic components or collision with space debris are studied in two types of satellites: cubesats and microsats. Low Earth Orbit (LEO) case studies are proposed, and the level of catastrophic failure occurrence is quantified for the same mission duration. The variation in the occurrence of failures is studied and there is a determination of which is more likely. Emphasis is placed on examining how an increase in space debris would affect the reliability of future space missions.

## Introduction

Space environment is a branch of astronautics, aerospace engineering and space physics concerned with understanding and addressing existing conditions in space that affect the design and operation of spacecraft. “Environmental effects on spacecraft can derive from radiation, space debris and meteoroid impacts, from atmospheric friction to high altitudes, and from electrostatic charging”^1–3^.

**Figure 1:**
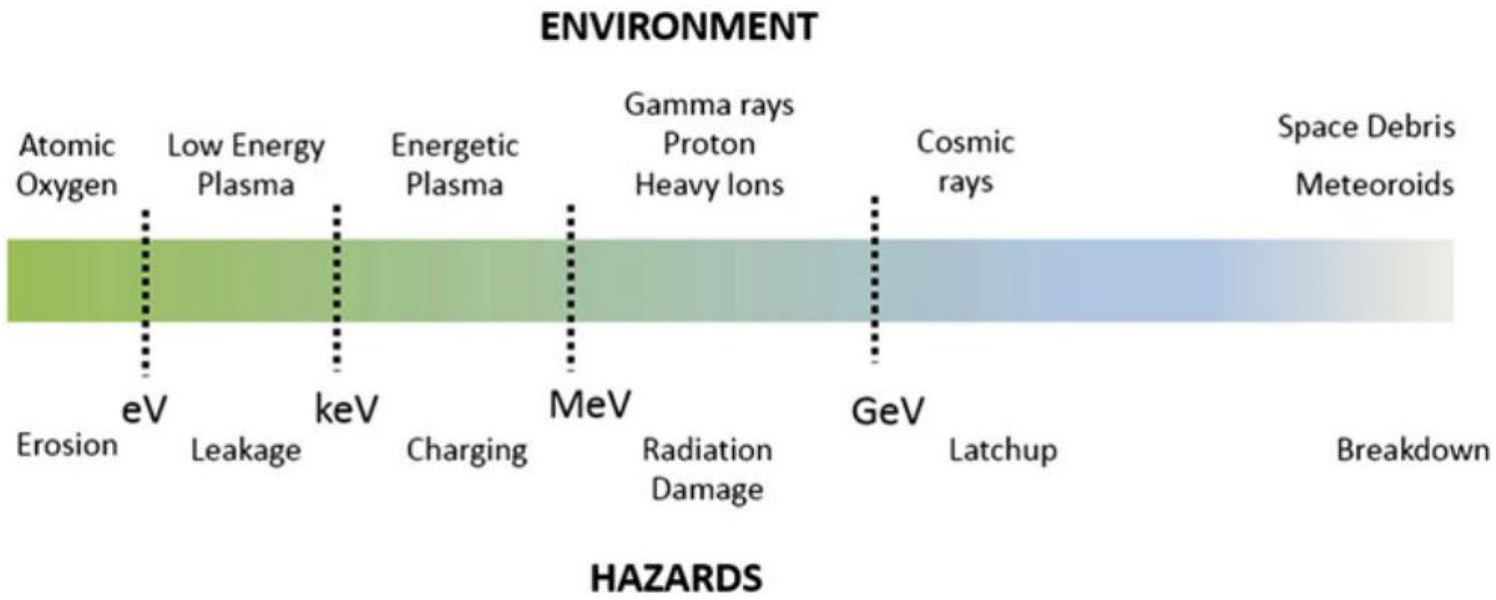
Space environment and hazards^1^.

A scale of the risks of the spatial environment in relation to the energy involved (eV) is shown in Fig. 1. The most energetic event in space is caused by small artificial residues or micrometeoroids. The impact of micrometeoroid or space debris can destroy a satellite. The second hazard is due to cosmic rays, which are sources of ionizing radiation. Cosmic rays are sufficiently energetic to cause “soft errors” in onboard electronic components, such as data corruption in RAM as well as poor processor performance, or “hard errors”, meaning destructive failures such as latch-up, which can produce destruction of electronic components and subsequent propagation through the system, resulting in a catastrophic failure for the entire mission^1,4^.

**Figure 2:**
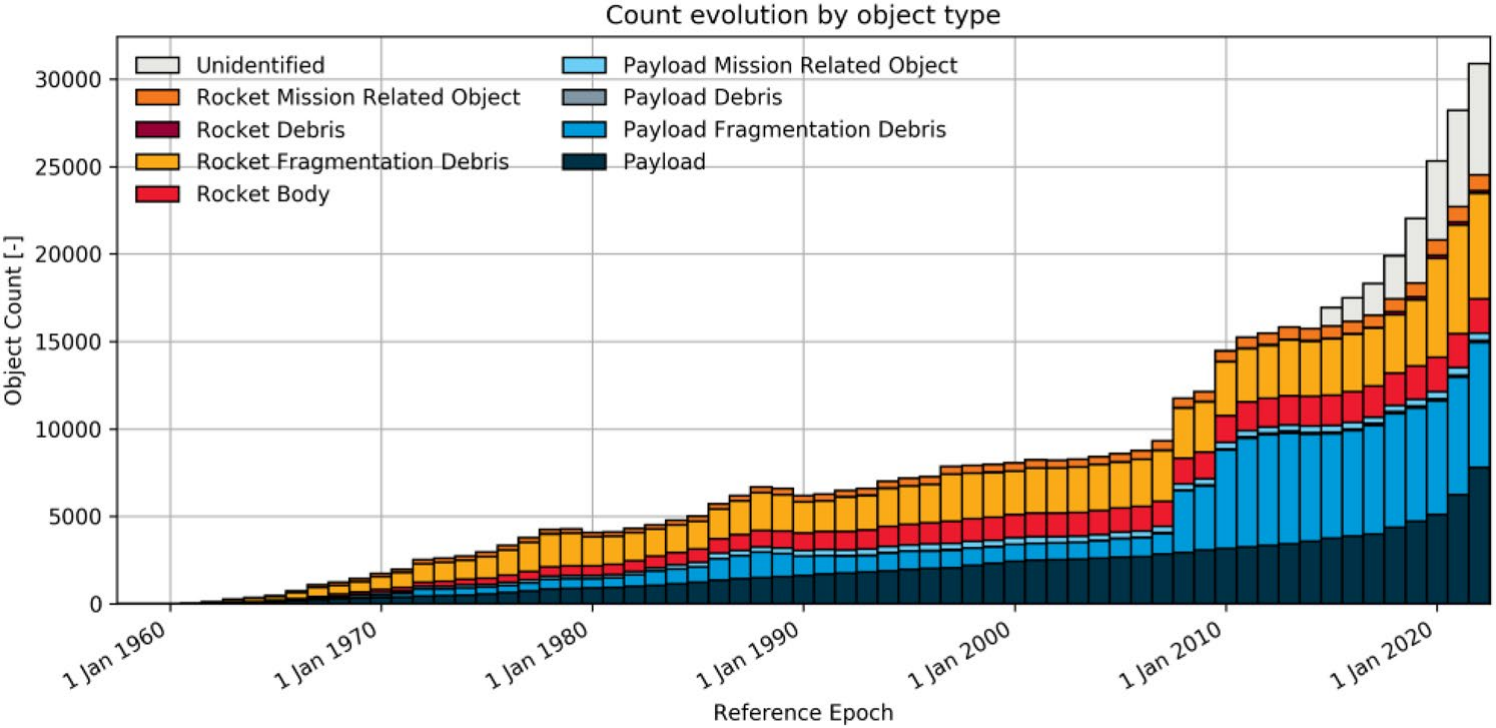
Evolution of space environment in terms of number of objects^5^.

Since the space environment population data show how the object count is increasing year by year (Fig. 2), our aim is to quantify the probability of catastrophic space debris collision compared with catastrophic failure by radiation effects for the same spacecraft, orbit, and mission duration.

Our purpose is to discover if the probability of catastrophic failures due to debris collision throughout the mission period is lesser, equal, or greater than the probability of catastrophic failures due to radiation effects, which has been considered since the eighties with a consolidated list of Radiation Hardness Assurance (RHA) standards^6,7^.

In this way, we are conducting a significant comparative study on how important the risk of debris collision is compared to the consolidated RHA methodology to improve mission reliability, with the aim of helping the space community and the space sectors answer this question: “How important space debris collision hazards are becoming in relation to the reliability of our satellites?”. The population of space debris is increasing with the “New Space” breakthrough, and answering this question can help the community to comprehend how important this is becoming in order to understand what we are facing with the debris population increase.

Finally, our goal is to bring a comparative study to the space community to promote the necessary stakeholder participation in space debris mitigation policy investments and provide some inputs to ensure mission reliability.

## LEOs under study

**Figure 3:**
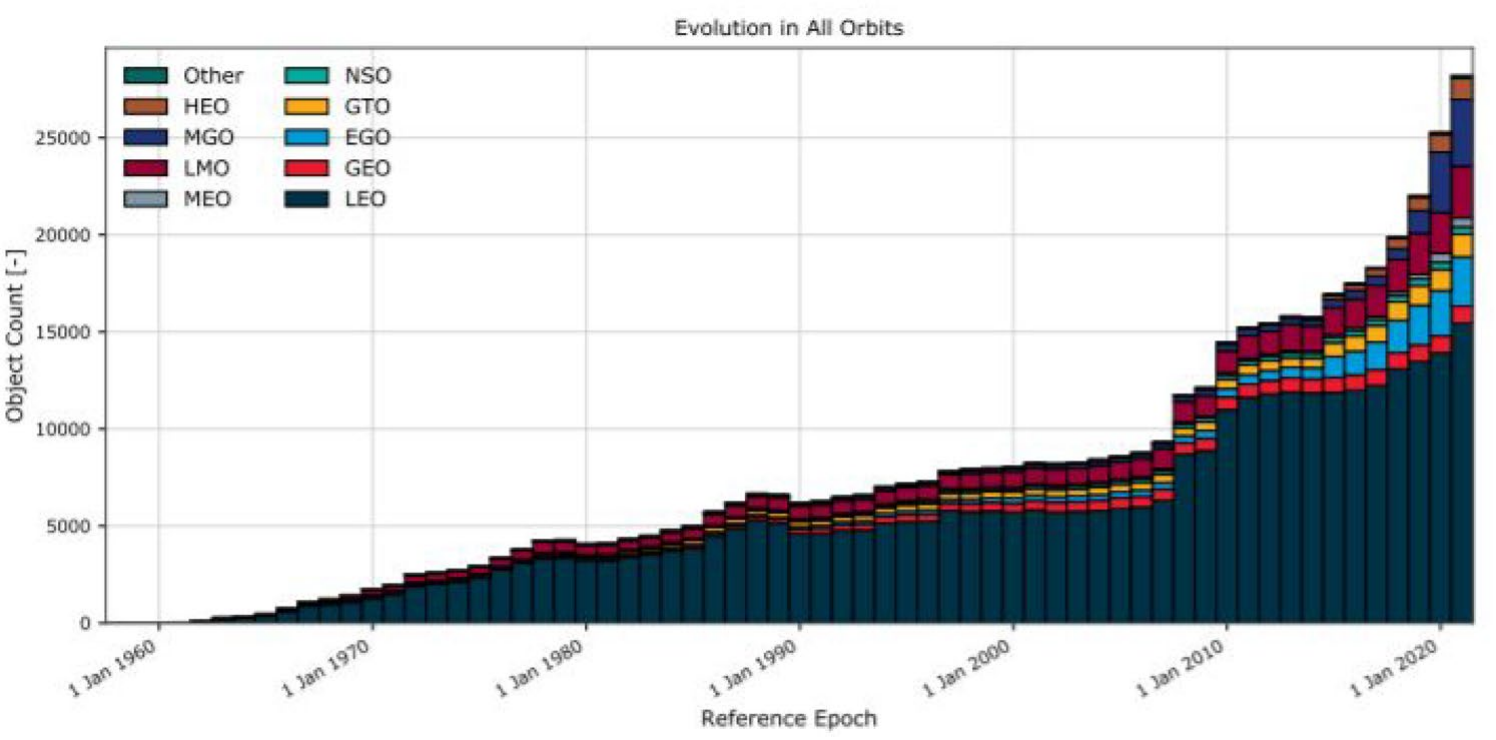
Evolution of number of objects per orbit altitude^5^.

LEOs have been chosen because they are the most popular orbits for the “New Space” cubesat and microsat market (see Fig. 3). It is clear that there is a greater number of objects in LEO than in others orbits.

**Figure 4:**
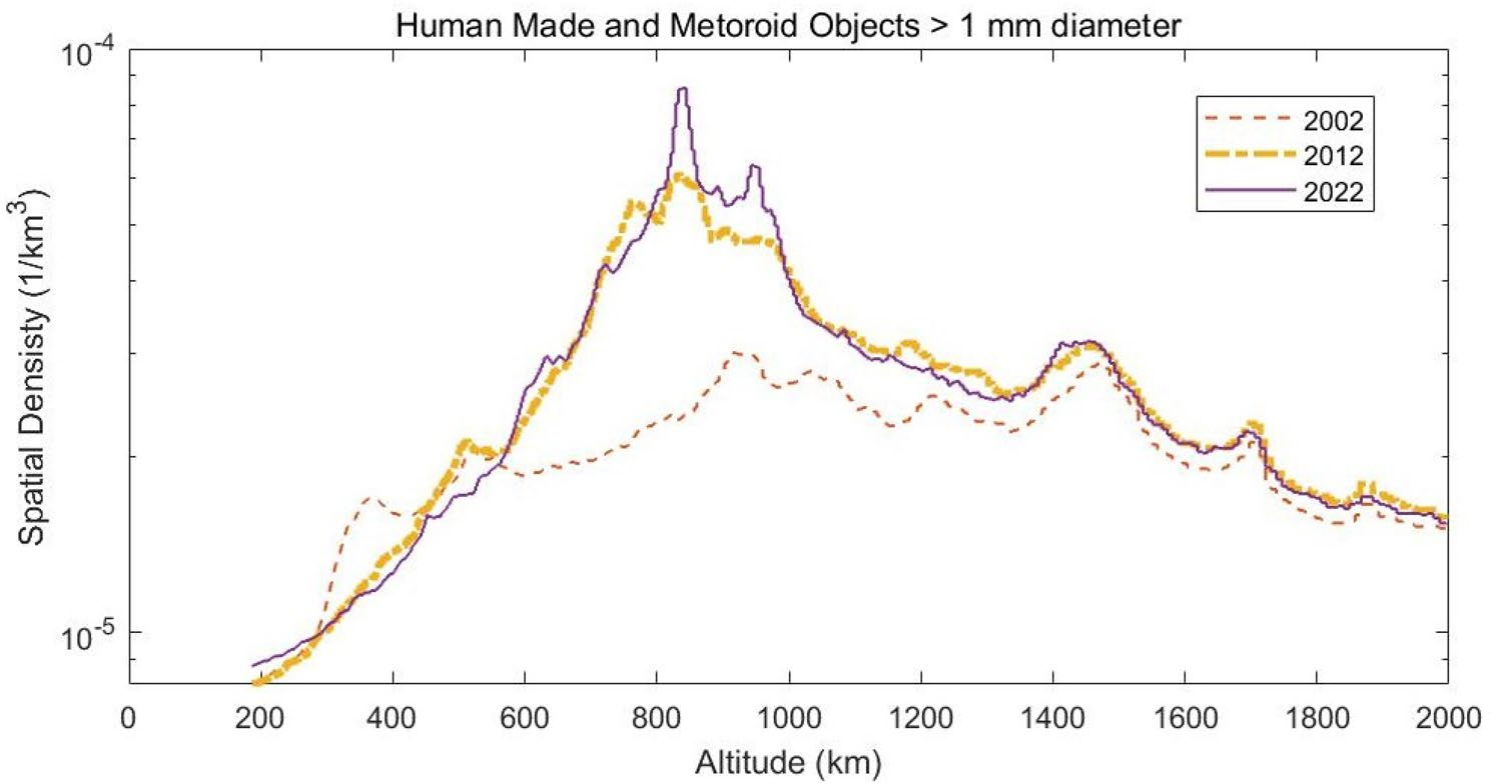
Human made and meteoroid spatial density objects with diameter higher than 1 mm. ESA-MASTER Model v8.0.2.

Within LEOs, those in our study have more population or ’hot spots’ as described in Fig. 4: both the 840 km and 960 km altitude, and the 407 km orbit for the International Space Station (ISS). A short description of their representativeness is given below:

- LEO-ISS is equal to that of the International Space Station (ISS). This is located in the LEO region at an altitude of approximately 407 km. Given it has an inclination of $51^{\circ }$, this mission does not orbit around the terrestrial poles. Being in the LEO region, crossing the region of the Van-Allen belt (from an altitude of 200 to 400 km) and not crossing the poles, a mission with this orbit will not be greatly affected by the effects of radiation because it is under the protection of the terrestrial magnetic field.
- 840 km and 960 km LEOs are similar to the LEO-ISS but with a variation in mission altitude. This change in altitude places the mission within congested orbits which also have more orbital debris and radiation effects because of the increase in altitude. The high density of objects there, especially in comparison to the ISS orbit, is due to the significantly reduced influence of atmospheric drag, which has an otherwise cleansing effect. In addition to the existing number of satellites and second stages in these orbits, there have been two collision events which released a large amount of debris: the collision between the Cosmos-2251 (950 kg mass) and Iridium-33 (560 kg mass) satellites in 2009^8^ and the intentional breakup of Fengyun-1C on 11 January 2007 anti-satellite test resulting in a complete destruction of the Fengyun-1C satellite (mass of 880 kg)^9^. Due to the fact that cubesats or microsats are usually inserted at these altitudes in sun-synchronous orbits (sso), the orbit of 840 km has been studied with a $98^{\circ }$ inclination.

Within LEOs, the main parameters for the 3 orbit simulations are described in Table 1.

**Table 1:** Main mission parameters for the study.

LEO orbit	407 km (ISS)	840 km-sso	960 km
Begin date	2022/01/01	2022/01/01	2022/01/01
Disposal phase	2022/06/30	2022/12/31	2022/12/31
Semi-major axis (km)	6778	7211	7731
Eccentricity	0.001	0.001	0.001
Inclination (deg)	51.5	98	51.5
RAAN (deg)	0.0	0.0	0.0
Argument of Perigee (deg)	0.0	0.0	0.0

## Radiation effects on EEE components

Radiation effects on EEE (Electrical, Electronic and Electromechanical) parts fall roughly into three categories: cumulative degradation from both total ionizing dose (TID) and total non-ionizing dose (TNID) and transient effects provoked by single charged particles, called single event effects (SEE)^5^.

Due to TID and TNID being cumulative effects and requiring long-duration missions to cause a radiation effect, in the following we are going to only take into account the probability of a hard SEE event occurrence along a mission duration of 1 year, the essential number to compare with the probability of catastrophic event occurrence by space debris collisions for the same mission duration.

**Figure 5:**
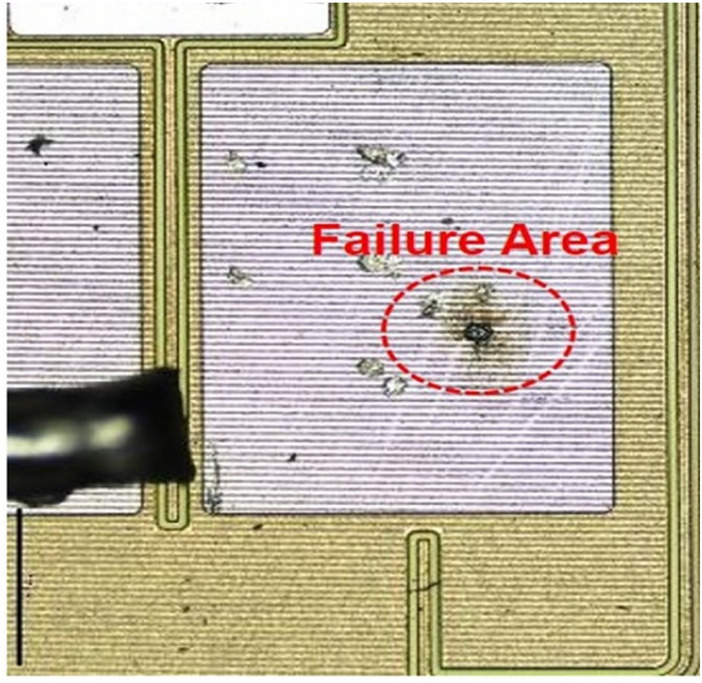
Hard SEE-SEB (single event burn-out) microphotography^10^.

Transient SEEs are the result of particle ionization as they pass through a sensitive surface of the electronic device. SEEs are caused by heavy ions but protons can also contribute^1,5,7^. Some SEEs are non-destructive and are known as “soft errors”, with others being destructive and called “hard errors”. Most hazards for entire system reliability are the well known hard errors: SEL (Single Event Latch-Up), SEGR (Single Event Gate Rupture), and SEB (Single Event Burn-Out). The SEB effect is shown in Fig. 5 to illustrate how a cosmic ray has provoked the ignition by SEB occurrence in the electronic part^10^. SEB occurs during triggering of the parasitic bipolar structure in a power transistor, accompanied by regenerative feed-back, avalanche and high current condition. SEB is potentially destructive unless properly protected against.

The preferred method for dealing with destructive failures is to use “Rad-Hard” parts, and when SEE-hard parts are not available, latch-up protection circuitry can be employed in conjunction with in-depth failure mode analysis and RHA methodology for the system design.

To describe the radiation effects on electronic devices, the concept of linear energy transfer (LET) is used. The robustness of the device is determined for the LET value that causes SEE. The SEE mission environment specification for a spacecraft is the documentation of the particle flux (number of particles passing through a unit area per second) versus LET spectra^5^.

The instantaneous ionization of a particle through the medium is measured by its LET. The electronic stopping power or linear energy transfer (LET) is the amount of energy lost by the incident ion along its path in the absorber medium when colliding with atomic electrons. LET is expressed in units of energy per unit length, MeV/cm divided by the mass density of the absorber medium; therefore, LET is expressed in units of MeV cm$^{2}$/mg.

The SEE sensitivity of an electronic component is expressed by its cross section $(\sigma )$ in Eq. (1), which means the number of events per unit fluence, expressed in units of cm$^{2}$/device or cm$^{2}$/bit

1 $$\begin{aligned} \sigma =\frac{N_{events}}{\phi } \end{aligned}$$

where fluence $(\phi )$ is the total number of particles/cm$^{2}$ hitting the device along the mission duration, which is obtained by multiplying particle flux ($\hbox {p}/\hbox {cm}^2 \, \hbox {s}$) by mission duration in seconds. Assuming that SEE events are random, cross section is the probability of events. This comparative study associates a COTS (commercial of-the-shelf) part with a saturation cross-section of $\sigma =10^{-4}$ cm$^{2}$/device or cm$^{2}$/bit, so it is necessary for around 10,000 particles to hit the device in order to induce a catastrophic error, meaning high SEE radiation sensitivity. In addition, a Rad-hard part is associated with a saturation cross section of $\sigma = 10^{-6}$ cm$^{2}$/device or cm$^{2}$/bit, it is necessary for around 1 million particles to hit the device to induce a catastrophic error, which means low SEE radiation sensitivity. These saturation cross section values have been chosen to be sufficiently representative for the different radiation tolerance of electronic devices and to have a strong differentiation of 2 orders of magnitude between Rad-Hard part (very low radiation sensitivity) and COTS part (high radiation sensitivity). The authors would note that radiation-tolerant electronic devices have a cross-section lower than $\sigma = 10^{-7}$ cm$^{2}$/device or cm$^{2}$/bit, as described in the ESCC Basic Specification No. 25100 Single Event Effects Test Method and Guidelines^11^, but many manufacturers sell their devices as radiation-tolerant or radiation-hardened with cross-section values higher or lower than this reference value. It is up to the satellite operator to choose a particular threshold that is most representative based on the sensitivity of the onboard electronics. Knowledge of parts radiation sensitivity is an essential aspect of the overall RHA program. It could be useful to highlight that many small satellites include COTS parts instead of rad-hard ones, due to cost saving and a more risk-oriented approach.

### OMERE software for estimating catastrophic SEE failure rate probability

The use of “Space Weather” prediction software to discover the single particle environment flux vs LET spectrum of a space mission is widely extended. One such preferred software package for radiation engineers is the OMERE program^12^.

Ionizing particle fluence for a mission $\phi$ (which appears in Eq. 2) depends on orbit, launch date and mission duration. We consider the LEO typical mission duration as being 1 year, except for LEO (ISS) which lasts 6 months due to its orbit decay beyond this limit because of friction forces and not propulsion. The launch date was 1 January 2022. Radiation prediction or space weather forecast for Single Event Effects has been computed with OMERE choosing the following standard prediction models, based on the requirements defined according to ECSS-E-ST-10-04 standard^13^.

- Cosmic ions: ISO 15390 model
- Solar particles average: ESP (emission of solar protons) for protons and Psychic model for heavy ions with 95% confidence level and solar fare model: none for protons
- Heavy ions (HI): CREME96 WC (worst case) 1 day model.

“This standard applies to all product types which exist or operate in space and defines the natural environment for all space regimes. It also defines general models and rules to determine the local induced environment”^13^.

There is a representation of the particle flux versus LET for the ISS, 840 km-sso and 960 km orbits under study during the year 2022. Computed simulation results show how SEE radiation levels are constant over time and mostly equal for all LEO orbits. Simulations show the same radiation levels for the current year 2022, one decade before (year 2012), and one decade after (year 2032). This is because rather than depending on launch time, radiation levels depend mostly on mission duration and orbit parameters.

**Figure 6:**
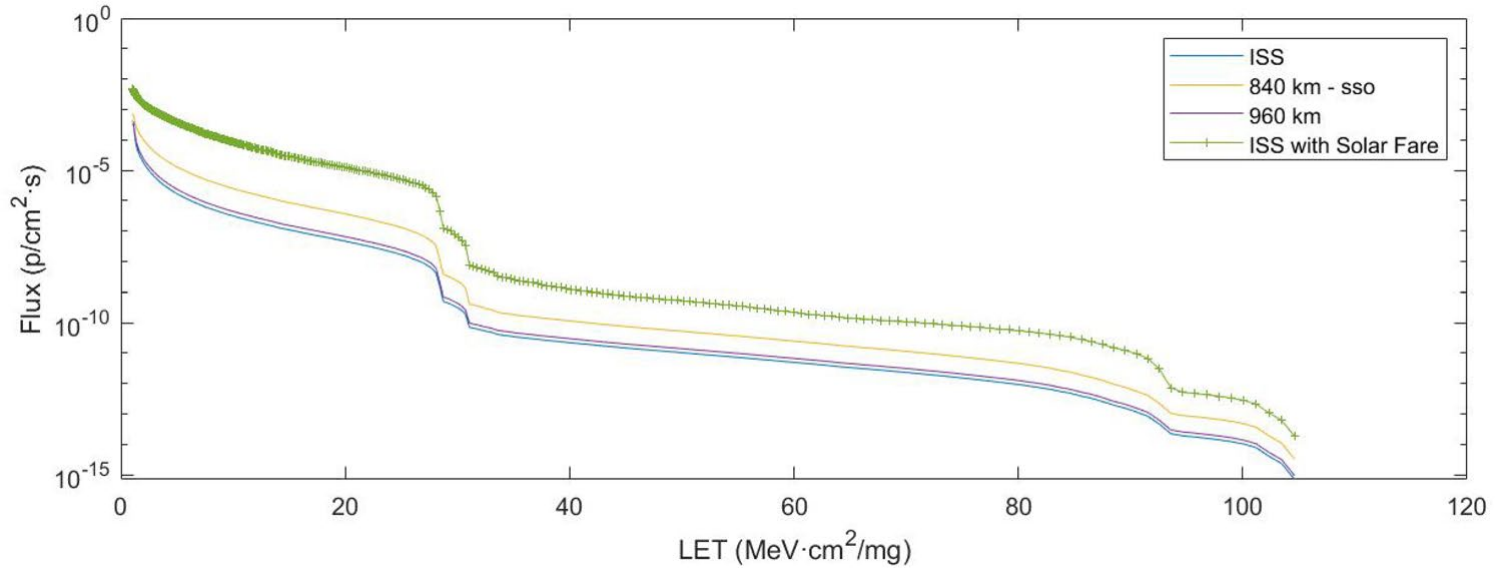
Particle flux per LET values computed with the OMERE software for each orbit in 2022.

In addition, the ISS orbit is also represented but takes into account a solar flare in Fig. 6. Radiation levels are highly dependent on solar flares, but until now there is no tool to predict this significant event. It is clear that if a solar flare or CME (coronal mass ejection) occurs, the probability of catastrophic failure provoked by radiation increases by 2 orders of magnitude and can clearly be the root cause of the fatal failure. Thus, for this reason, we are not planning on undertaking this study during a solar flare, and we are providing a tool to evaluate what kind of event is more likely to occur considering standard space environment conditions for satellite operation.

**Figure 7:**
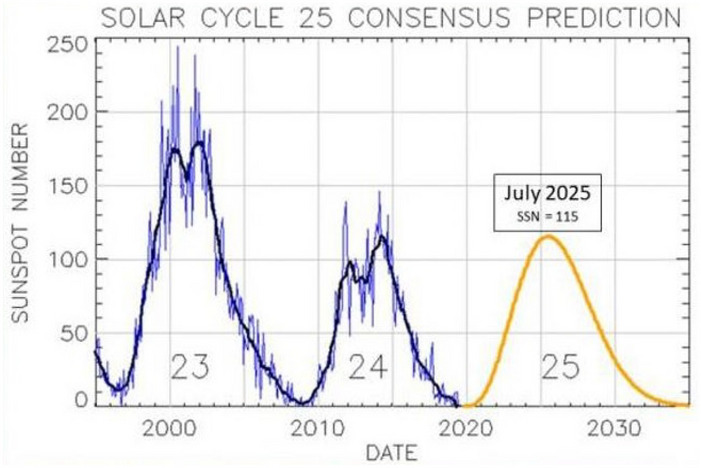
The NOAA/NASA co-chaired, international panel to forecast Solar Cycle 25 released their latest forecast for Solar Cycle 25. ©NOAA Space Weather Products and Services.

Moreover, our study covers the year 2022, which is between the minimum and maximum of solar cycle 25 (see Fig. 7), related to an increase in solar activity (sun spots, solar flares and CME). 2022 is therefore an important representative year for satellite operation in normal conditions. The aim of the study is to capture the most common scenario, not the worst or best case (minimum and maximum solar cycle). In this sense, the year 2022 represents a common scenario because is in between min and max of solar cycle 25.

The data show how there is no high dependence on orbit altitude, as all orbits are considered as LEOs. Differences in radiation levels are observed with MEO (Medium Earth Orbit) and GEO (Geostationary Earth Orbit) because of the higher altitudes and the atmosphere reduction. There is also a dependence on the orbit latitude with the radiation levels, because of the Earth’s magnetic field.

### Hard errors or catastrophic failures provoked by SEE

In order to calculate the probability of SEE catastrophic failure we have taken into account the total particle fluence with an LET value high enough to produce a catastrophic error on the part. Considering that LET values are between 1 and 110 MeV cm$^{2}$/mg (with higher fluxes at lower LET values), we have defined a LET threshold value above 15 MeV cm$^{2}$/mg to produce a catastrophic error and therefore LET below 15 MeV cm$^{2}$/mg can only produce soft errors in on-board electronics or, at least, there is a very low probability that they will do so compared to LET values above 15 MeV cm$^{2}$/mg.

In Table 2 there is a representation of the cumulative particle fluxes as a function of the minimum LET threshold value for the different orbits studied. It can be seen how the fluxes with different LET values are 3 orders of magnitude higher/lower than the flux for LET values higher than 15. Once again, the satellite operator will be in charge of estimating the lowest LET threshold of the electronic parts on board to estimate their radiation sensitivity. For this study, we have taken into account a LET threshold of 15 because it is the most representative probability and it is not an extreme case as described in Table 2. Cumulative particle flux with LET >1 is too high and with LET>30 it is too low.

**Table 2:** Cumulative particle flux (p/cm$^2$ s) for different orbits and LET threshold.

LEO orbit	$\sum _{LET=1}^{LET=110} Flux$	$\sum _{LET=15}^{LET=110} Flux$	$\sum _{LET=30}^{LET=110} Flux$
407 km (ISS)	7.89E$-$03	2.65E$-$06	2.14E$-$09
840 km-sso	2.44E$-$02	2.09E$-$05	1.27E$-$7
960 km	1.01E$-$02	3.73E$-$06	2.92E$-$09

Assuming only one sensitive electronic part is on board, following Eq.(1) and the assumptions described above, we compute the occurrence of a catastrophic event provoked by radiation in the following equation.

2 $$\begin{aligned} N_{Catastrophic-events}= {\sigma \cdot \phi _{LET>15}} \end{aligned}$$

Following the above criteria (see Eq. 2), the results of the OMERE simulations are reported in Table 3. In addition, there is a color code representation for their associated risk level according to their Qualitative Failure Probability (QFP), which is defined as an example of the occurrence levels and limits of the levels^14^. Thus, the color of the cell is related to their associated QFP, which is defined as $QFP=1-e^{-N}$, where N is the number of catastrophic events defined in Eq. (2).

**Table 3:**
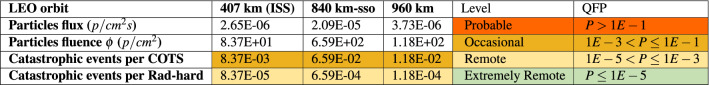
Catastrophic events caused by radiation for computed orbits with OMERE software.

## Satellite impact with space debris and meteoroids

The primary sources of the impacting particles are the Earth-orbiting population of space debris and the meteoroid complex. While the meteoroid complex is the natural result of our Solar System’s dynamic dust balance, the cloud of space debris in Earth-orbit is produced artificially in the process of exploration and commercialization of the near-Earth environment^15–17^.

There is a description of the orbit object population as a function of time in Fig. 3 which shows how LEO orbits now have the highest increasing object population, corresponding to the irruption of “new space”: small satellites with lower launch costs than in the past. Due to the fact that the Inter-Agency Space Debris Mitigation Coordination Committee (IADC) defines ’space debris’ as ’all man-made objects including fragments and elements thereof, in Earth orbit or reentry into the atmosphere that are nonfunctional’, there is an intrinsic relationship between the most occupied orbits and the space debris population, meaning that LEO has the potential to be the highest risk for space debris collision. This is because the main orbits in our study are those with more population or “hot spots” as described in Fig. 4: 840 km and 960 km altitude LEOs.

### Kinetic of catastrophic collisions provoked by space debris and meteoroids

As shown in ESA handbook^18^, the main parameter to determine whether a collision is catastrophic, i.e., generates fragments, is the energy-to-mass ratio (EMR), which has been defined in Eq. (3):

3 $$\begin{aligned} EMR=\frac{(1/2) \cdot M_{p}\cdot V_{imp}^{2} }{M_{t}} \end{aligned}$$

where $\hbox {M}_{\rm{p}}$ is the mass of the projectile (that is, the orbiting and uncontrolled debris), $\hbox {V}_{\rm{imp}}$ is the impact velocity (that is, the relative velocity between the projectile and the target), $\hbox {M}_{\rm{t}}$ is the mass of the target.

The threshold for a catastrophic collision (EMR)$_{{\textrm{cc}}}$ is assumed to be 40 J/g, and $\hbox {V}_{{\textrm{imp}}}$ is around 20 km/s (typical velocity value), as described in the ESA Handbook^18^. Thus, it is possible to extract a threshold mass for the projectile as described in Eq. (4):

4 $$\begin{aligned} M_{p}=\frac{2\cdot EMR_{cc}\cdot M_{t} }{V_{imp}^{2}} \end{aligned}$$

To convert this threshold mass to a threshold size, a standard projectile with the following characteristics should be used

- Area-to-mass ratio: ($\hbox {A}_{\rm{p}}$/$\hbox {M}_{\rm{p}}$)std = 0.01 m$^{2}$/kg
- Spherical surface with diameter D: $\hbox {A}_{\rm{p}} = \pi /4\cdot D_{p}^{2}$

Following this criterion, a catastrophic collision has occurred if the diameter of projectile $D_{p}$ is defined as follow^18^

5 $$\begin{aligned} D_{p}\ge \sqrt{\frac{8}{\pi }\cdot \frac{A_{p}}{M_{p}}\cdot \frac{EMR_{cc}\cdot M_{t} }{V_{imp}^{2}}} \end{aligned}$$

The diameter of the projectile for producing a catastrophic event, $D_{p}$, as a function of mission parameters: mass and cross-sectional area for a cubesat or microsat, has been calculated and reported in Table 4. It can be seen that this projectile diameter is equal to 1.6 mm for a cubesat and 1.13 cm for a microsat.

**Table 4:** Satellite parameters.

	Cubesat	Microsat
Cross-sectional area /m$^2$	0.01	0.25
Mass/kg	1	50
Dp/m	0.0016	0.0113

**Figure 8:**
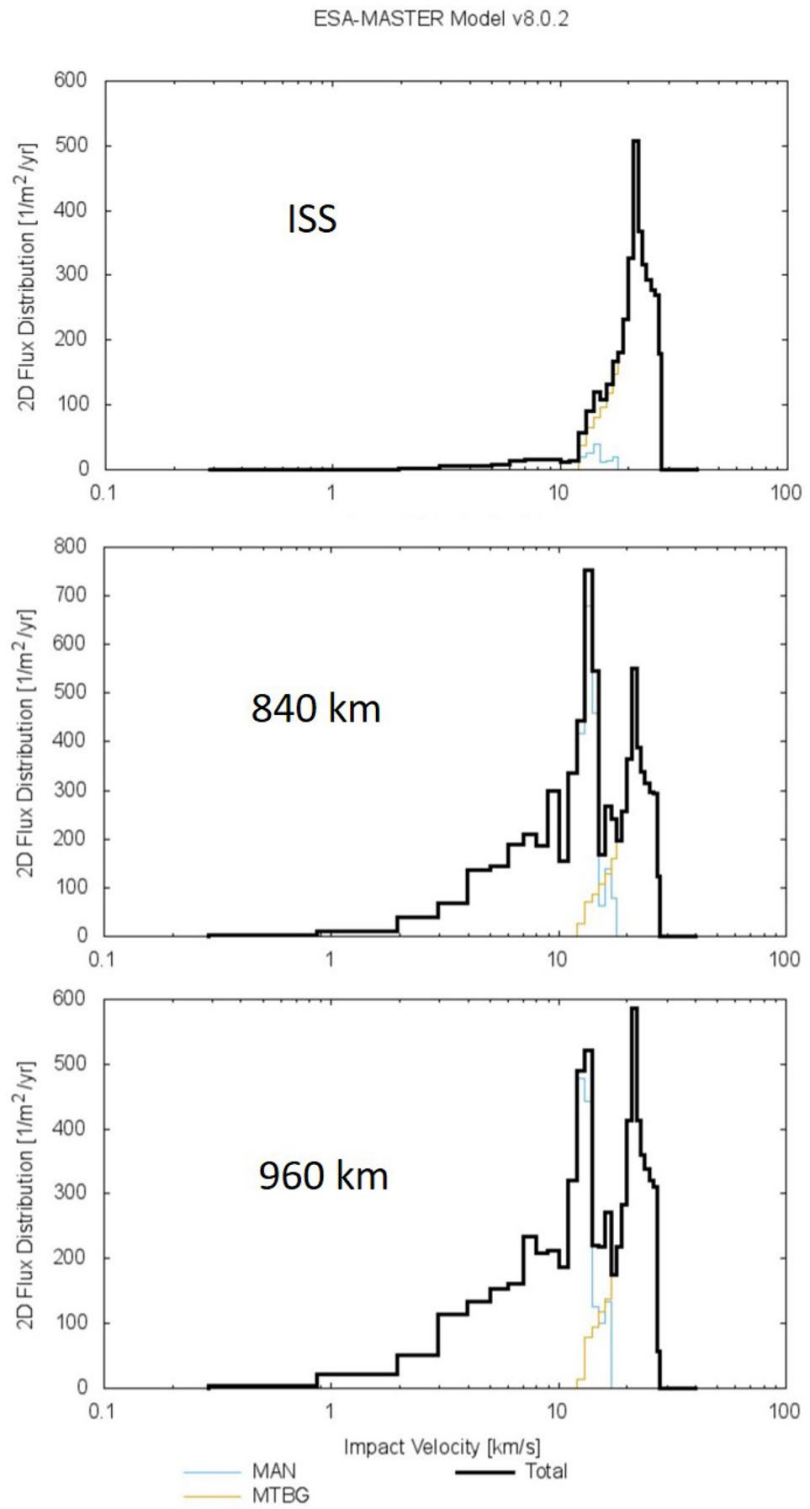
Impact Velocity as a function of different orbits calculated with DRAMA^20^ software.

Note than $\hbox {EMR} = 40 \,\hbox {J/g}$ and $\hbox {Vimp} = 20 \,\hbox {km/s}$ have been chosen as typical values because they are reported in different articles^11,19^. Moreover, the impact velocity as a function of the orbit can also be extracted with the software DRAMA^20^ (Debris Risk Assestment and Mitigation Analysis). The typical velocity for the objects placed in the orbits under study can be seen in Fig. 8. The maximum typical input velocity is between 10 and 20 km/s.

With the purpose of noting what differences would be implied by changing the EMR values or impact velocity in the Dp values, we have counted the differences in the fluxes as a function of these parameters in Tables 5 and 6.

**Table 5:** Particle fluxes as a function of Dp for a cubesat.

EMR	Vimp (km/s)	Dp cubesat (m)	ISS (p/cm$^2$ s)	840 km-sso (p/cm$^2$ s)	960 km (p/cm$^2$ s)
20	20	1.13E$-$03	9.95E$-$06	1.93E$-$04	1.16E$-$04
20	15	1.50E$-$03	4,72E$-$06	–	–
40	20	1.60E$-$03	2.30E$-$06	–	–
60	20	1.95E$-$03	2.30E$-$06	–	–
40	15	2.13E$-$03	1.20E$-$06	–	–
20	10	2.26E$-$03	1.20E$-$06	–	–
60	15	2.61E$-$03	1.20E$-$06	–	–
40	10	3.19E$-$03	4.40E$-$07	–	–
60	10	3.91E$-$03	4.40E$-$07	2.39E$-$05	6.87E$-$06

**Table 6:** Particle fluxes as a function of Dp for a microsat.

EMR	Vimp (km/s)	Dp cubesat (m)	ISS (p/cm$^2$ s)	840 km-sso (p/cm$^2$ s)	960 km (p/cm$^2$ s)
20	20	7.98E$-$03	1.33E$-$06	1.07E$-$04	5.50E$-$05
20	15	1.06E$-$02	4.72E$-$06	–	–
40	20	1.13E$-$02	2.30E$-$06	–	–
60	20	1.38E$-$02	2.30E$-$06	–	–
40	15	1.50E$-$02	1.20E$-$06	–	–
20	10	1.60E$-$02	1.20E$-$06	–	–
60	15	1.84E$-$02	1.20E$-$06	–	–
40	10	2.26E$-$02	4.40E$-$07	–	–
60	10	2.76E$-$02	1.43E$-$07	2.31E$-$05	7.50E$-$06

Dp values have been placed from low to high as a function of EMR and Vimp. It can be observed that a small variation in these parameters, from 20 to 60 in case of EMR and from 10 to 20 in case of Vimp, affect the results by one order of magnitude in particle flux as maximum, therefore this is the maximum error variation of our calculations for the final conclusions. The authors wish to give an overview of the implications of choosing general parameters. One of the aims is to have an outline of the root cause for a catastrophic event so that knowing its occurrence probability can be help it to be determined. Once again, the satellite operator can conduct this study as soon as the assigned mission parameters are known to better evaluate the ballistic limit of their structures.

### DRAMA software for estimating catastrophic collision failure rate probability

**Figure 9:**
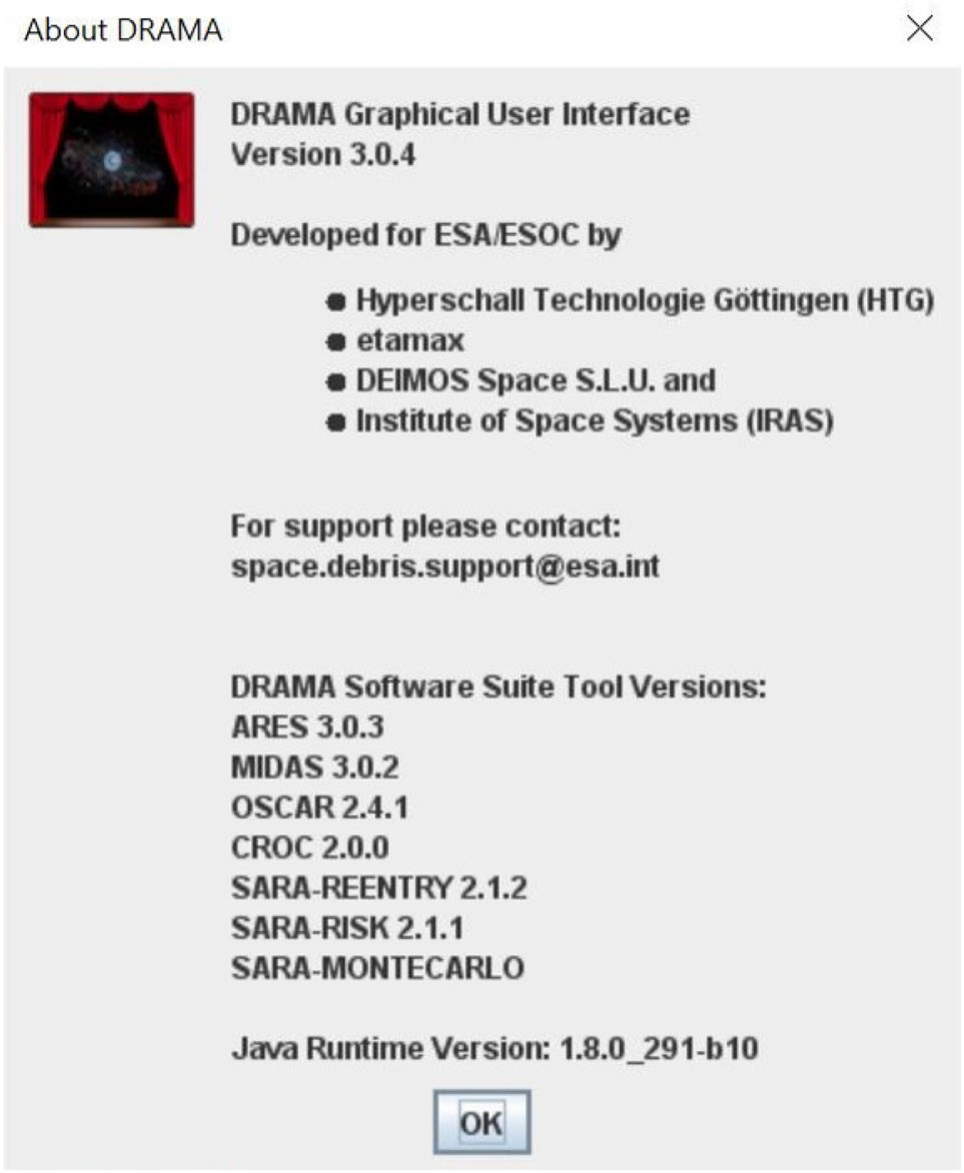
Information about DRAMA and MIDAS version which has been used for the simulations^20^.

In order to calculate the number of catastrophic collisions for a mission, we use DRAMA software (see Fig. 9) to count the cumulative flux for all diameters larger than the critical diameter computed via Eq. (5). DRAMA software contains many tools for different purposes. In our case, we have used the Micro-Imaging Dust Analysis System (MIDAS) tool to compute our simulations with the DRAMA software. MIDAS studies the dust environment around asteroids and comets, providing information on particle population, size, volume and shape.

The DRAMA simulations results are reported in Table 7. The ISS orbit shows an extremely remote Qualitative Failure Probability (QFP) while high altitude LEO orbits show the same QFP level as the rad-hard part QFP level.

**Table 7:**
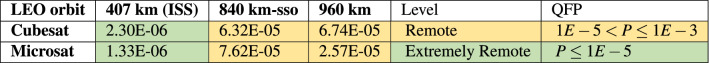
Catastrophic events caused by debris collisions for cubesat and microsat missions with DRAMA software.

Another very interesting result is the variation in QFP as a function of satellite mass and surface area, that is, for a cubesat or a microsat. We have taken into account cubesats and microsats because they are the most commonly employed types of satellite in LEO for the new space market. Furthermore, a cubesat with more than 1 unit can be considered a microsatellite.

Results show how the QFP level is mostly equal at 840 km but is slightly higher for the cubesat at 960 km. Although this result can be considered as a contradiction due to the high surface impact of the microsat, it is explained because the cumulative flux of particles with a diameter higher than corresponding Dp values reported in Table 4. The cumulative flux of particles with Dp> 1.13 cm diameter in a 960 km orbit is lower than the cumulative flux of particles with diameter Dp> 1.6 mm that is required for the cubesat to produce a catastrophic collision.

In terms of reliability impact, and following this conclusion, it is recommended that a satellite be flown with a slightly higher surface area and mass than a cubesat in the 960 km orbit. A larger satellite can also be better tracked from the ground (and thus collision avoidance will work more effectively), and a larger area and/or mass allows for better shielding design, etc.

## Results and comparative analysis

**Figure 10:**
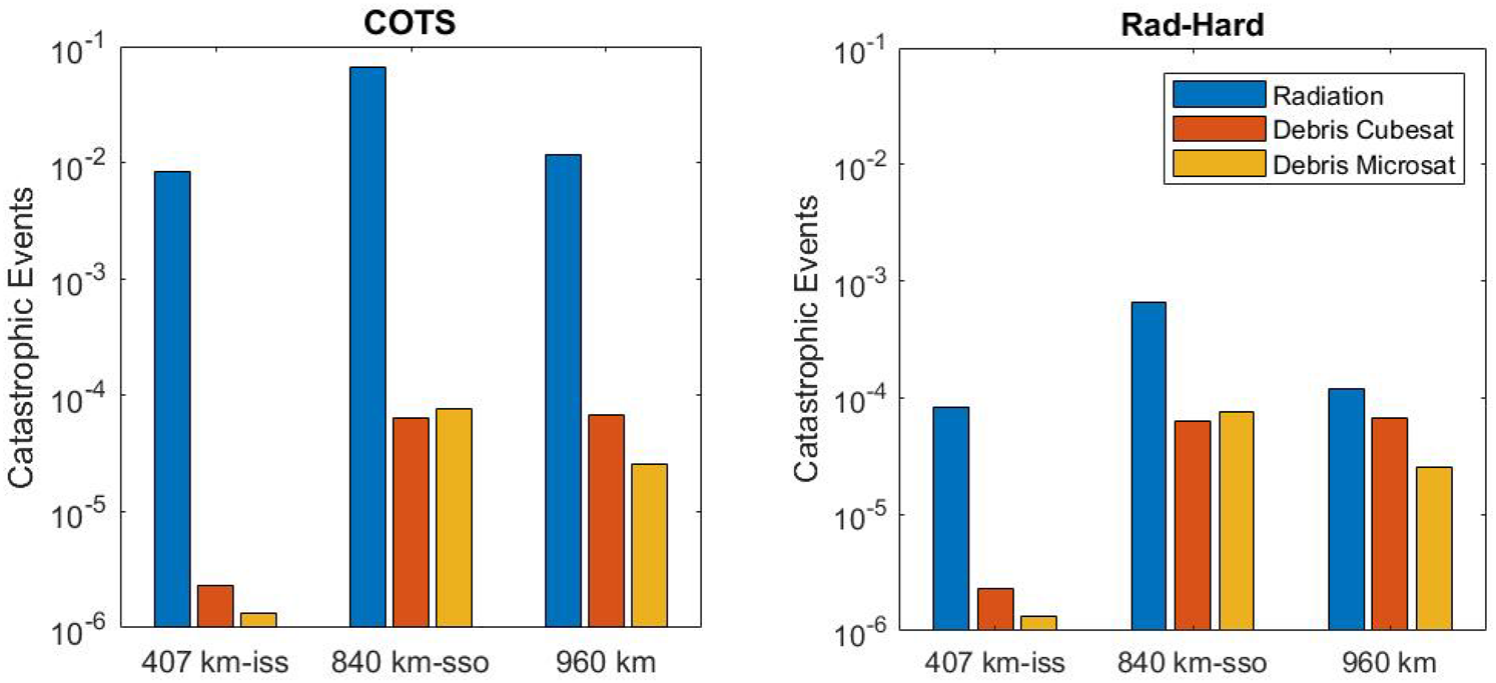
Catastrophic event occurrence by radiation or debris collisions for cubesat and microsat at LEO orbits in 2022.

The radiation and debris results have been plotted together in Fig. 10 to compare the difference for its occurrence level for the entire year 2022. The QFP for radiation is greater for the ISS orbit because of its lower debris population than in high altitude orbits, however, it can be seen how catastrophic events by radiation is only 2 order of magnitude higher than debris collision at higher orbits. In addition, if a spacecraft has been designed with Radiation Hardness Assurance (RHA) methodology or has rad-hard parts on board, the occurrence level for debris collision is the same of that for radiation effects for the 960 km orbit. Therefore, in the event of a catastrophic error, it will be difficult to determine whether the root cause was a radiation effect on electronics or a debris collision.

In addition, the study includes some of the results that will occur in the near future. Figure 4 shows the evolution over time of debris and meteoroid population with a diameter of 1 mm or over. Since meteoroids can be considered mostly constant over time, it is clear that human-made debris has been increasing from 1957 (first satellite launched) to the present day. Moreover, the spatial density of debris has experienced more rapid growth from 2012 to 2022 than in the previous decade.

**Figure 11:**
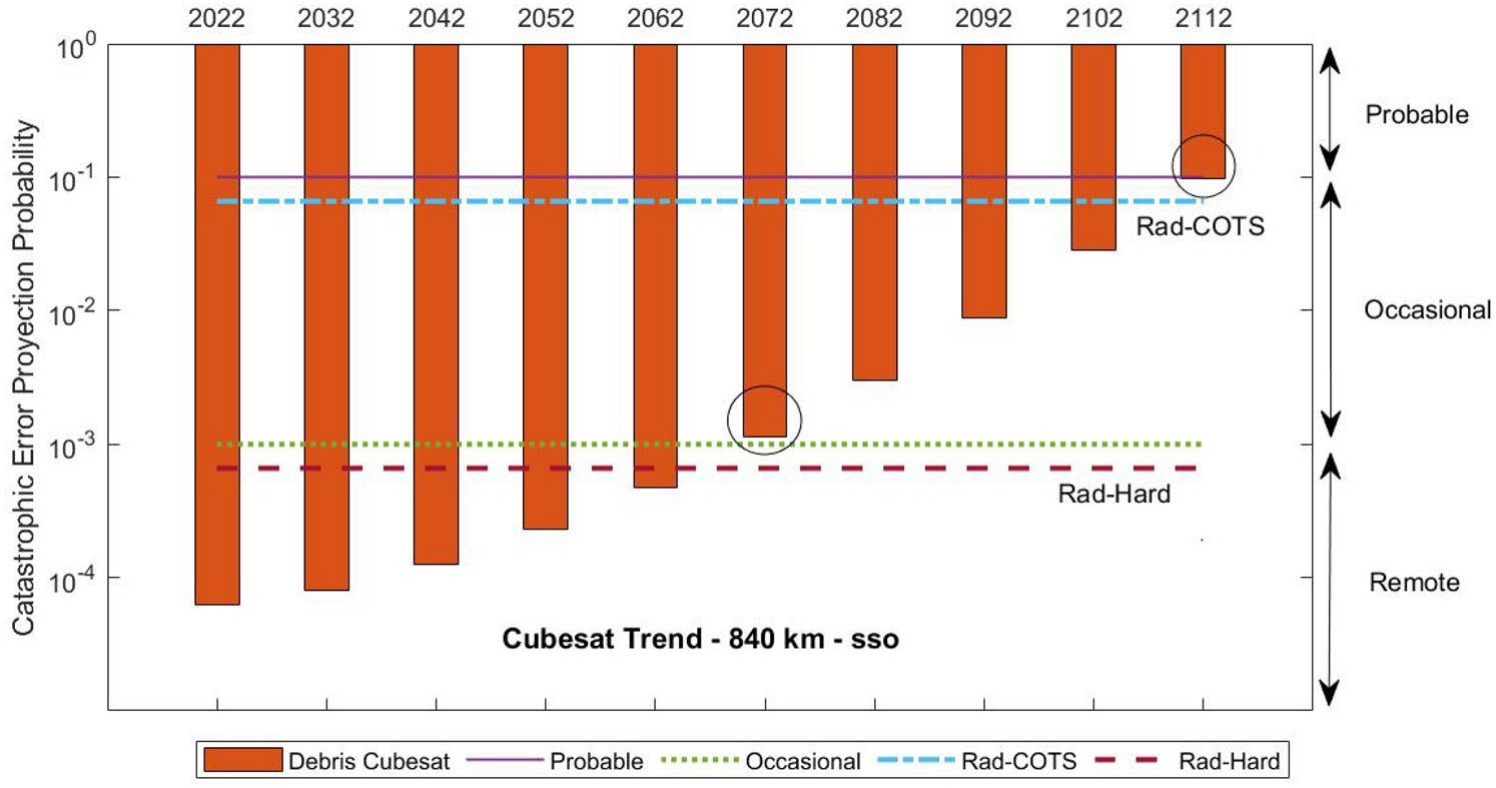
Cubesat risk level according to failure probability in time due to debris collision or radiation effects at 840 km–sso LEO orbit.

**Figure 12:**
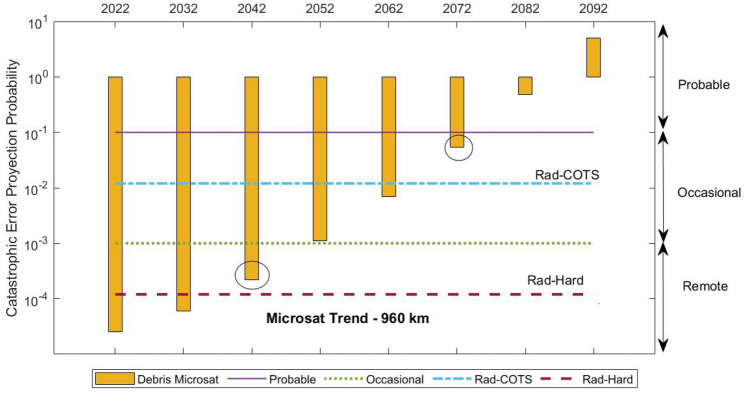
Microsat risk level according to failure probability in time due to debris collision or radiation effects at 960 km altitude LEO orbit.

This problem is alluded to in Figs. 11 and 12. Taking into account the quantity of debris population that has grown in the 840 km-sso orbit or 960 km orbit since the last decade, we plot this constant debris increment for the next 100 years. It can be seen how the QFP projection by a debris collision will overcome the QFP for radiation effects on cubesats with hard parts in just 50 years. In addition, space debris collisions will be more likely than radiation events in less than a century. However, the microsat trend shows a rapid overcoming of the debris impact risk, only in 50 years from now, even with COTS parts on-board.

The foregoing means that spatial density debris may become a further problem with the same magnitude as natural radiation if there are no policies to remove debris or minimize the generation of new space debris.

## Conclusions

Through this research paper we have highlighted the impact of space debris collisions on the reliability of space satellites in the context of the new space market.

By this comparison, we are offering the space community a quantitative tool to compare the impact of space debris collisions with the well-known impact of radiation effects on the reliability of components and systems in LEO orbits.

The study results conclude that radiation-related risks will no longer be the first level of possibility in less than 50 years from now in LEO orbits. On the contrary, the space collision risk will prevail over the intrinsic risk that spacecraft encounter with respect to their operation in a natural space radiation environment.

Our study also shows a maximum error/variation of one order of magnitude as a function of the parameters variations involved in the simulations. It can be seen in Fig. 12 that a variation in 1 order of magnitude implies an error margin of 2 decades. Thus, the main conclusion that debris-induced reliability issues will predominate radiation effects in 50 years from now can be, expanded to a range of between 50 and 70 years, with a high probability of success in the prediction.

Projection of this study has been made considering a constant increment of debris population over the following years, instead of another kind of trend that may be more likely to occur as a consequence of the fast growth of the “new space” market or the application of new polices for debris reduction and final mission disposal. This indicates that a high degree of uncertainty may exist and would be part of any future analysis. In this way, we are able to understand how important the implications of space debris are and to force the application of a policy of space debris reduction in order to alter the trend and minimize its impact.

On the assessment of the root cause, the authors could invite the readers (e.g. spacecraft designers) to think more about this. A debris or meteoroid impact is likely to change the spacecraft attitude, while radiation wouldn’t. The chain of actions after impact or radiation absorbing leading to catastrophic error might be different depending on where it hits. Space debris would preferably hit from the front direction (in flight direction). These are points that could be mentioned, which allow to still establish what might have been the more likely root cause, even if they appear on the same order of magnitude given this analysis.

## Data Availability

All data generated or analyzed in this study are included in this article. Published data can be generated by any user with the software and steps described in the article. In any case, the data sets used or analyzed in the current study are available for the corresponding author on reasonable request.
